# Near-infrared spectroscopy-derived tissue oxygen saturation in battlefield injuries: a case series report

**DOI:** 10.1186/1749-7922-4-25

**Published:** 2009-06-19

**Authors:** Greg J Beilman, Juan J Blondet

**Affiliations:** 1 Division of Surgical Critical Care/Trauma, Department of Surgery, University of Minnesota. MMC 11. 420 Delaware St SE. Minneapolis, Minnesota 55455, USA

## Abstract

**Background:**

Near-infrared spectroscopy technology has been utilized to monitor perfusion status in animal models of hemorrhagic shock and in human traumatic injury. To observe the effectiveness of such a device in a combat setting, an FDA-approved device was used in conjunction with standard resuscitation and therapy of wounded patients presenting to the 228^th ^Combat Support Hospital (CSH), Company B, over a three-month period.

**Materials and methods:**

These observations were performed on patients presenting to the 228^th ^CSH, Co B, at Forward Operating Base Speicher, outside of Tikrit, Iraq, between the dates of June 15 and September 11, 2005. We utilized the Inspectra™ 325 tissue oxygen saturation (StO_2_) monitor (Hutchinson Technology, Inc; Hutchinson, MN, USA) with the probe placed on the thenar eminence or on another appropriate muscle bed, and used to monitor StO_2 _during early resuscitation and stabilization of patients.

**Results:**

During the above time period, 161 patients were evaluated at the CSH as a result of traumatic injury and the device was placed on approximately 40 patients. In most patients, StO_2 _readings of greater than 70% were noted during the initial evaluation. No further information was collected from these patients. In 8 patients, convenience samples of StO_2 _data were collected along with pertinent physiologic data. In these patients, StO_2 _levels of below 70% tracked with hypotension, tachycardia, and clinical shock resulted in increases in StO_2 _after resuscitation maneuvers.

**Conclusion:**

Near-infrared spectroscopy-derived StO_2 _reflected and tracked the resuscitation status of our patients with battlefield injuries. StO_2 _has significant potential for use in resuscitation and care of patients with battlefield injuries.

## Background

Optimal treatment for early hemorrhagic shock includes adequate control of bleeding followed by restoration of tissue oxygen delivery with appropriate resuscitation. Unfortunately, from a military perspective, this optimal strategy may not be available for many patients due to field situations that preclude prompt transport to the appropriate treatment facility [1]. Therefore, determination of the magnitude of shock using a rapid, non-invasive method may be useful at the point of care in the field in both military and urban trauma settings. Such a method has the potential to be of use for appropriate triage depending on availability of medical resources.

**Figure 1:**
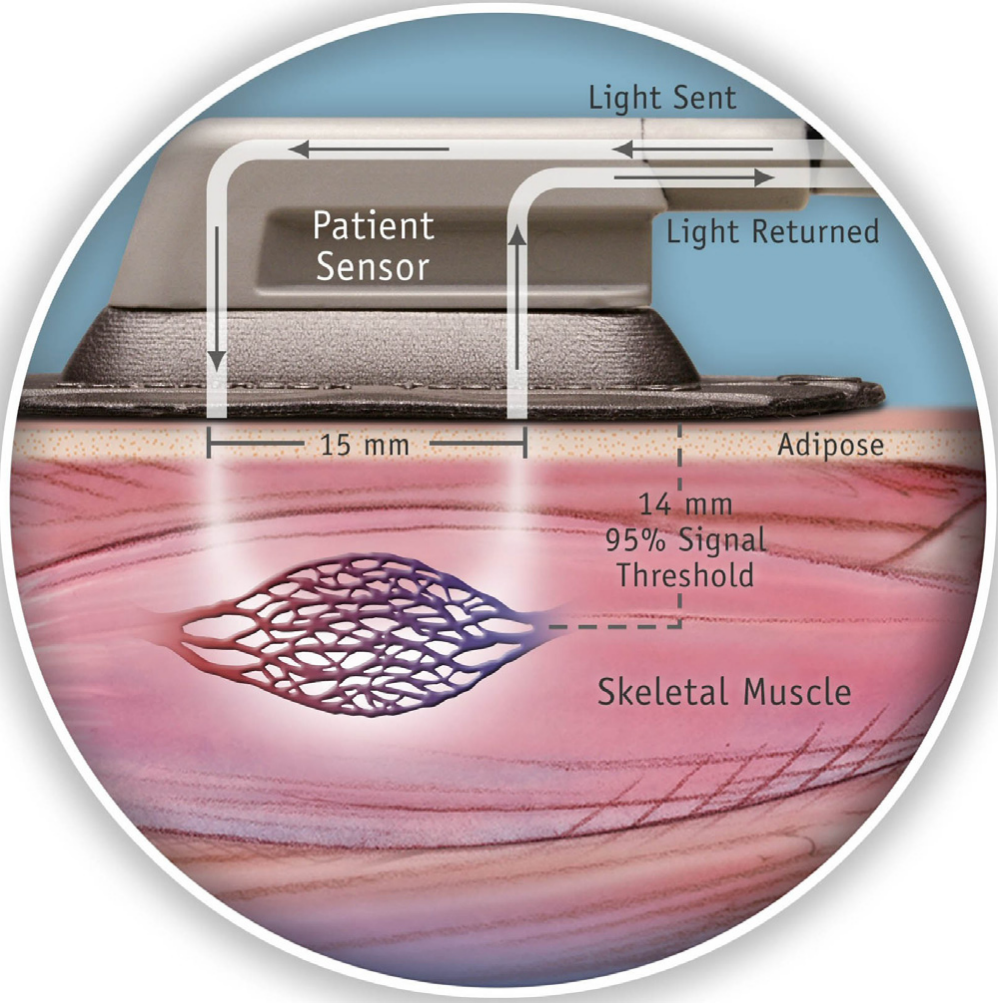
**StO_2 _is derived from measurement of the near-infrared spectra of the tissue bed sampled**. A near-infrared light source shines light into the tissue bed. A spectrum, measured using reflectance of near-infrared light, is used to measure the percentage of hemoglobin saturation.

Near-infrared (NIR) spectroscopy utilizes fiber-optic light to non-invasively determine the percentage of oxygen saturation of chromophores (e.g. hemoglobin) based on spectrophotometric principles [2]. This technology has been utilized to experimentally determine regional tissue oxygen saturation (StO_2_) [3-5] by monitoring the differential tissue optical absorbance of near-infrared light. Unlike pulse oximetry, NIR spectroscopy measures not only arterial, but also venous oxyhemoglobin saturation at the microcirculatory level (Figure 1). This measurement therefore is a reflection of both oxygen delivery (DO_2_) and oxygen consumption (VO_2_) of the tissue bed sampled [6,7]. Non-invasive determination of these parameters using NIR spectroscopy has been described as has its correlation with DO_2 _and mixed venous oxygen saturation (SvO_2_) [3-7]. NIR-derived StO_2 _has been demonstrated to be predictive of severity of shock states in an animal model of hemorrhagic shock [8].

To observe the effectiveness of such a device in a combat setting, an FDA-approved device was used in conjunction with standard resuscitation and therapy of wounded patients presenting to the 228^th ^Combat Support Hospital (CSH), Company B, over a three-month period.

## Materials and methods

**Figure 2:**
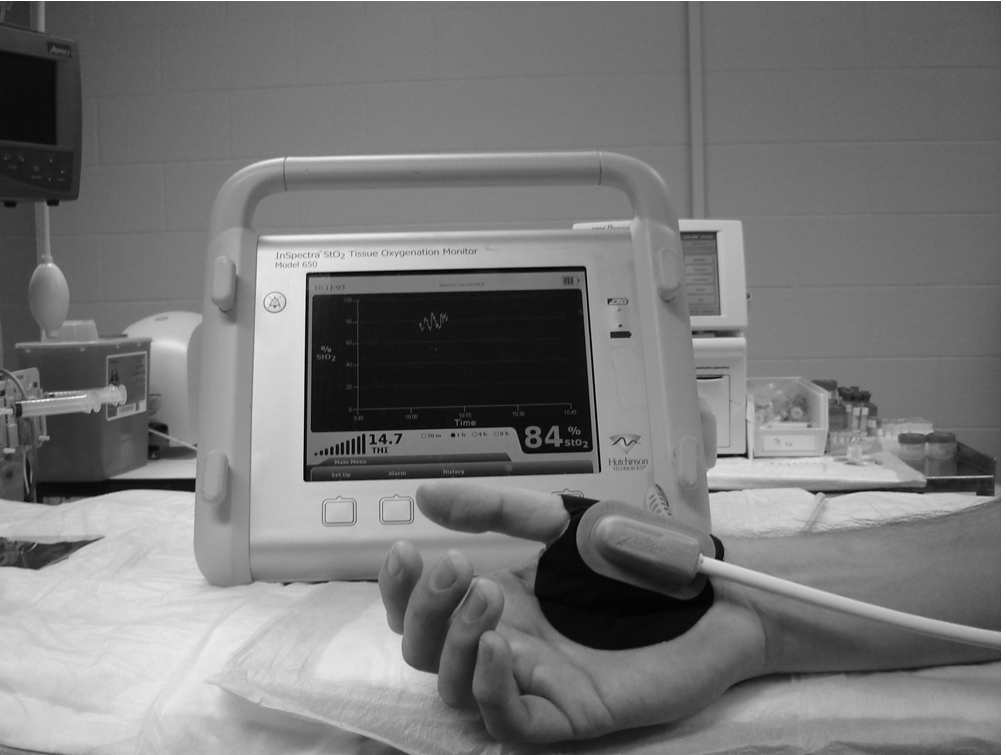
**The non-invasive StO_2 _probe is placed directly over the thenar eminence of the patient**. The device will continuously generate StO_2 _readings every 4 seconds.

These observations were performed on patients presenting to the 228th Combat Support Hospital (CSH), Company B, at Forward Operating Base Speicher, outside of Tikrit, Iraq, between the dates of June 15 and September 11, 2005. These observations were performed during use of the Inspectra™ 325 as a clinical monitor (Figure 2). The Brooke Army Medical Center Institutional Review Board waived the need for informed consent. The Inspectra™ StO_2 _tissue oxygenation monitor (Hutchinson Technology, Inc; Hutchinson, MN, USA) is currently FDA-approved for use in monitoring patients continuously during circulatory or perfusion examinations of skeletal muscle, or when there is a suspicion of compromised circulation. A recent large observational and descriptive study found a mean thenar StO_2 _of 87 ± 6% in 707 normal human volunteers [9]. In the present observations, a 70% cutoff value of StO_2 _was selected to screen for patients to be followed in time because data obtained from severely injured trauma patients has verified that a StO_2 _value of less than 75% is predictive of multiple organ failure and mortality [10].

Patients were brought to the 228^th ^CSH via ground ambulance or helicopter after traumatic injury. Patients were evaluated by a team of physicians and health care providers using a standardized ATLS protocol and after stabilization taken as appropriate to the operating room and/or prepared for transfer to a higher level of care. Patients were monitored during resuscitation and early evaluation using clinical parameters, continuous EKG and pulse oximetry, and other monitors (e.g. bladder catheterization) as appropriate. In situations where more than one patient was evaluated concurrently, an attempt was made to place the StO_2 _monitor on the most severely injured patient. Convenience samples of demographic data, vital signs, laboratory data, and StO_2 _data were collected on patients as patient care permitted.

## Case presentations

Between June 15 and September 11, 2005, there were 161 patients evaluated at the 228^th ^CSH, Co B as a result of traumatic injury. The StO_2 _monitor was placed on approximately 40 patients during this period of time. In most patients, StO_2 _readings of greater than 70% were noted during the initial evaluation. No further information was collected from these patients. In 8 patients, convenience samples of StO_2 _data were collected along with pertinent physiologic data. In these patients, StO_2 _levels of below 70% tracked with hypotension, tachycardia, and clinical shock resulted in increases in StO_2 _after resuscitation maneuvers (Table 1). Four cases are presented in greater detail to illustrate the use of the device as correlated with patient status.

**Table 1:** Comparison of StO_2 _levels at presentation and after resuscitation maneuvers.

**Injury**	**Initial StO_2_**	**Resuscitation Maneuver**	**Post resuscitation StO_2_**
Bilateral lower extremity IED	60	2 LR, 2 PRBCs	78
IED blast, right leg, left flank	51	2 LR, 1 PRBCs	71
GSW left thigh	54	1 LR	88
Abdominal compartment syndrome	62	Open abdomen	91
Bilateral lower extremity IED	51	1 LR	76
GSW abdomen	50	1 LR	82
GSW right arm	55	0.5 LR (9 y/o)	76
Blast injury	1	CPR	1

Eight patients with StO_2 _levels measured at presentation and after initial resuscitation. LR: lactated ringers (expressed in liters); PRBCs: packed red blood cells (expressed in units); IED: improvised explosive device; GSW: gunshot wound; CPR: cardiopulmonary resuscitation.

### Case 1

A 36-year-old male was injured from an improvised explosive device (IED) and presented with near amputations of both lower extremities. He arrived at the emergency medical treatment area (EMT) with blood pressure (BP) of 110/70 mm Hg and heart rate (HR) of 120/min. His initial StO_2 _reading was 51% from the right thenar eminence. He received 1 liter of lactated ringers (LR) with an increase in StO_2 _to 76% and was taken to the operating room (OR) where he underwent a right below the knee amputation and debridement and external fixator placement for a complex left tibia fracture.

The next morning, the patient's StO_2 _was noted to be low at 40%. His BP was 105/72 mm Hg and HR was 130/min with hemoglobin of 8.9 g/dl. Over the next 2 hours, the patient received 300 cc of 25% albumin, 1 liter of LR, and 1 unit of packed red blood cells (PRBCs) with HR decreasing to 110/min, and BP increasing to 130/70 mm Hg, and urine output of 150 cc over the previous hour. StO_2 _increased to 73%.

This patient's post-injury course was long and complicated. After multiple operations including debridements and skin grafting, the patient was discharged from the hospital approximately 2.5 months after his initial injury.

### Case 2

A 24-year-old male was seen in the EMT after a gunshot wound (GSW) to the abdomen. His initial vital signs included a BP of 90/60 mm Hg and HR of 120/min. His initial StO_2 _from the thenar eminence was 50%. He received 1 liter of LR with an increase of his BP to 110/70 mm Hg and StO_2 _to 82%. He was taken to the OR where he was found to have a tangential transverse colon injury. He underwent a primary repair and recovered and was discharged from the hospital approximately 2 weeks post-injury.

### Case 3

A 20-year-old male presented to the EMT after a high-velocity GSW to the left hip. At the time of presentation, two peripheral intravenous (IV) lines, which had been placed in the field, were infiltrated. One wound was noted in the left lateral hip and the patient had a distended, tense, and tender abdomen. His initial BP was 56/30 mm Hg and HR was 150/min. Arterial oxygen saturation (SaO_2_) was 100% and thenar StO_2 _was 54%. A left subclavian line was placed and patient received 1 liter of crystalloid with a response of BP to 110/70 mm Hg and HR to 120/min. His StO_2 _increased to 88%.

He was taken to the OR where exploratory laparotomy and repair of small bowel enterotomies was carried out. Proctoscopy was negative. He received 4 units of PRBCs and 2500 cc of crystalloid in the OR. His postoperative vitals were BP of 110/68 mm Hg, HR of 100/min, SaO_2 _of 100% and StO_2 _of 89%. Two hours later, he became hypotensive and oliguric and StO_2 _decreased to 65%. He received 2 liters of crystalloid, 2 units of fresh frozen plasma (FFP), and 1 unit of PRBCs with an improvement of BP, urine output, and StO_2 _(82%).

**Figure 3:**
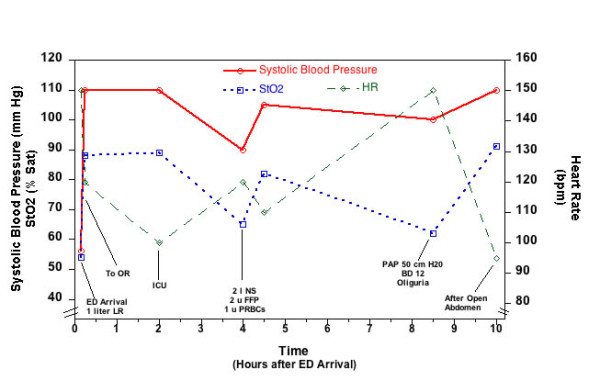
**Graphic representation of systolic blood pressure, heart rate, and StO_2 _of patient described in case 3 during the first 10 hours of hospital course**.

Approximately 8 hours after the patient's initial presentation he developed recurrent oliguria, increased airway pressures (Peak pressures of 50 cm H_2_O with tidal volumes of 6 cc/Kg). His BP was 100/60 mm Hg and HR of 150/min with a base deficit of 12 mEq/L. StO_2 _had dropped to 62%. The patient was taken to the OR where his abdomen was opened and a Bogota bag was placed with immediate improvement of all parameters (StO_2 _increased to 91%). (Initial hospital course: Figure 3)

His post-injury course was complicated and included development of necrotizing muscle infection, internal iliac arterial bleed, and ureteral fistula requiring left nephrectomy. He was eventually discharged from the hospital 3 months after his injury.

### Case 4

A 36-year-old male suffered an IED injury resulting in a massive injury to the right lower extremity. He was hypotensive in the field with a systolic BP (SBP) of 77 mm Hg. A tourniquet was placed and the patient was transferred via air to our facility. He arrived at the EMT with a SBP of 69 mm Hg, HR of 150/min, SaO2 of 91%, and StO_2 _of 51%. In the ED he received 2 liters of LR and 1 unit of O negative PRBCs with an improvement of his vital signs and StO_2 _(SBP 110 mm Hg, HR 125/min, StO_2 _71%). Initial injuries noted included left pulmonary contusion, open right femur fracture, large soft tissue injury in left buttocks, and laceration of the right radial artery.

He was taken to the OR where the tourniquet was removed and injuries to the profunda femoral artery and vein were noted. Multiple branches were ligated and oversewed. The sciatic nerve and superficial femoral artery were both intact. The patient had massive soft tissue injury that was widely debrided. The shrapnel in his left buttocks was removed (proctoscopy was negative). He developed coagulopathy, an external fixator was placed, and the patient was returned to the intensive care unit (ICU) for further resuscitation (INR: 10, platelets: 33,000, and hemoglobin: 3.9 g/dl). During his OR course the patient's StO_2 _dropped to 51% just prior to transfer to the ICU. His final OR temperature was 36.6°C. OR fluids included 13 liters of crystalloid, 4 units of FFP, and 9 units of PRBCs.

On arrival in the ICU, the patient's initial SBP was 82 mm Hg, HR 130/min, and StO_2 _50%. Initial hemoglobin was 7.9 g/dl and base deficit was 16 mEq/L. Over the next 4 hours the patient received 9 units of FFP, 10 mg of vitamin K, 2 units of fresh whole blood, 4 units of PRBCs, 200 cc of 25% albumin, 2 liters of LR, and 6500 mcg of Factor VIIa. Two hours into the resuscitation 2 plateletpheresis packs arrived via helicopter and were given. With this therapy the patients' vital signs and urine output improved gradually (BP 100/70 mm Hg, HR 90/min, and urine output 150 cc/hour) and his laboratory parameters likewise showed improvement with a normal INR, hemoglobin of 8.6 g/dl, platelets of 70,000/ml, and base deficit of 7 mEq/L. StO_2 _likewise slowly improved (65%).

**Figure 4:**
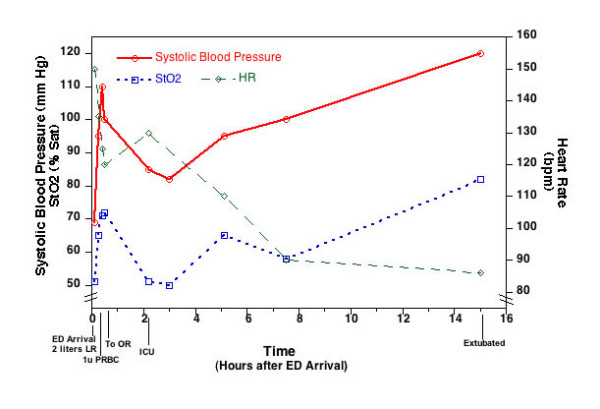
**Graphic representation of systolic blood pressure, heart rate, and StO_2 _of patient described in case 4 during the first 16 hours of hospital course**.

The next morning the patient was weaned and extubated. His platelet count and INR were normal. His StO_2 _was 82% (initial hospital course: Figure 4). He received debridement and progressive closure of his wound every other day and 10 days post-injury received intramedullary femoral rod for stabilization of his femur fracture. He was discharged from the hospital 24 days post-injury.

## Discussion

Care of patients in the austere environment of the battlefield presents challenges to the clinician, including limited access to invasive monitoring techniques readily available in the care of civilian trauma patient. Equipment utilized in a field situation must be readily transportable, rugged, reliable, and easy to use. Over the years, many technologies originally developed for civilian use have found their way into the armamentarium of battlefield care, including bedside ultrasound and computed tomography. Near-infrared spectroscopy has a similar promise for field use.

The patient experiences described above suggest that NIR spectroscopy-derived StO_2 _is able to serve as a non-invasive tool for early identification and treatment of hypoperfusion in the severely injured trauma patient. Nevertheless, in the present case series, the small number of patients described and the observational nature of this report preclude any generalization or formal recommendation.

A recent study of 383 trauma patients at 7 civilian trauma centers has identified the association of a low StO_2 _with both multiple organ failure and mortality [10]. There are currently no prospective studies examining its use as an endpoint for therapy in hemorrhagic shock. In the 8 patients described, StO_2 _followed the clinical course of the patient and in the 7 surviving patients tracked resuscitation status, suggesting that this measure may be potentially useful as such an endpoint. In addition, the readings from the StO_2 _monitor were more rapidly available and easier to categorize than other invasive or non-invasive hemodynamic tools used for determining need for additional resuscitation, and also had the advantage of easy interpretation regardless of the level or experience of the care provider. While there were no instances in this small series of abnormally low StO_2 _before clinical symptoms of shock were present, there is also the potential for such a device to be useful in early identification of "sub-clinical" shock.

Equally appealing is the possible use of StO_2 _in a triage setting in either civilian or military trauma. Such a use has the added benefit of giving a number to confirm the presence of tissue hypoperfusion for less experienced care providers. These potential benefits have led to the incorporation of StO_2 _as another tool for early evaluation of trauma patients at several civilian trauma centers.

Previous work from our lab in a porcine model of severe hemorrhagic shock identified StO_2 _as a significant predictor of eventual mortality in this setting [8], with StO_2 _significantly lower in the cohort of animals that were unsuccessfully resuscitated.

## Conclusion

Near-infrared spectroscopy-derived StO_2 _reflected and tracked the resuscitation status in the observed severely injured patients suffering battlefield injuries. StO_2 _has significant potential for use in resuscitation and care of patients with battlefield injuries.

## Competing interests

GJB has served on an Advisory Board and is the recipient of grant support from Hutchinson Technology, Inc. He is funded by the Office of Naval Research (#N00014-05-1-0344).

## Authors' contributions

GJB collected data from patients, collated data, and drafted the manuscript. JJB performed statistical analysis and coordinated manuscript preparation. All authors read and approved the final manuscript.

## About the authors

GJB serves as a Colonel in the United States Army Reserve. He's also Professor of Surgery and Anesthesia, Chief of the Division of Surgical Critical Care/Trauma, Vice Chair of Perioperative Services and Quality Improvement in the Department of Surgery at the University of Minnesota, and a Fellow of the American College of Surgeons. 

JJB served as a postdoctoral research associate at the Division of Surgical Critical Care/Trauma and currently is a general surgery resident in the Department of Surgery at the University of Minnesota.
